# Early mobilization of patients receiving extracorporeal membrane oxygenation: a retrospective cohort study

**DOI:** 10.1186/cc13746

**Published:** 2014-02-27

**Authors:** Darryl Abrams, Jeffrey Javidfar, Erica Farrand, Linda B Mongero, Cara L Agerstrand, Patrick Ryan, David Zemmel, Keri Galuskin, Theresa M Morrone, Paul Boerem, Matthew Bacchetta, Daniel Brodie

**Affiliations:** 1 Division of Pulmonary, Allergy, and Critical Care, Columbia University College of Physicians and Surgeons/New York-Presbyterian Hospital, New York, NY, USA; 2 Department of Surgery, Columbia University College of Physicians and Surgeons/New York-Presbyterian Hospital, New York, NY, USA; 3 Department of Medicine, Columbia University College of Physicians and Surgeons/New York-Presbyterian Hospital, New York, NY, USA; 4 Department of Clinical Perfusion, Columbia University College of Physicians and Surgeons/New York-Presbyterian Hospital, New York, NY, USA; 5 Department of Nursing, New York-Presbyterian Hospital, New York, NY, USA; 6 Department of Rehabilitation and Regenerative Medicine, New York-Presbyterian Hospital, New York, NY, USA; 7 Division of Thoracic Surgery, Columbia University College of Physicians and Surgeons/New York-Presbyterian Hospital, New York, NY, USA

## Abstract

**Introduction:**

Critical illness is a well-recognized cause of neuromuscular weakness and impaired physical functioning. Physical therapy (PT) has been demonstrated to be safe and effective for critically ill patients. The impact of such an intervention on patients receiving extracorporeal membrane oxygenation (ECMO) has not been well characterized. We describe the feasibility and impact of active PT on ECMO patients.

**Methods:**

We performed a retrospective cohort study of 100 consecutive patients receiving ECMO in the medical intensive care unit of a university hospital.

**Results:**

Of the 100 patients receiving ECMO, 35 (35%) participated in active PT; 19 as bridge to transplant and 16 as bridge to recovery. Duration of ECMO was 14.3 ± 10.9 days. Patients received 7.2 ± 6.5 PT sessions while on ECMO. During PT sessions, 18 patients (51%) ambulated (median distance 175 feet, range 4 to 2,800) and 9 patients were on vasopressors. Whilst receiving ECMO, 23 patients were liberated from invasive mechanical ventilation. Of the 16 bridge to recovery patients, 14 (88%) survived to discharge; 10 bridge to transplant patients (53%) survived to transplantation, with 9 (90%) surviving to discharge. Of the 23 survivors, 13 (57%) went directly home, 8 (35%) went to acute rehabilitation, and 2 (9%) went to subacute rehabilitation. There were no PT-related complications.

**Conclusions:**

Active PT, including ambulation, can be achieved safely and reliably in ECMO patients when an experienced, multidisciplinary team is utilized. More research is needed to define the barriers to PT and the impact on survival and long-term functional, neurocognitive outcomes in this population.

## Introduction

Neuromuscular weakness and the accompanying impairment in physical functioning are common sequelae of immobilization during critical illness [1-5]. Active participation in physical and occupational therapy is increasingly recognized as not only safe and feasible, but also the preferred approach to minimize debilitation in critically ill patients, including those who require invasive mechanical ventilation [6-8]. Patients who receive early rehabilitation have shown improved rates of returning to independent functioning, decreased rates of delirium, and shorter durations of mechanical ventilation, intensive care unit length of stay, and hospital length of stay [9-11].

Extracorporeal membrane oxygenation (ECMO) is increasingly being used in patients with respiratory failure [12,13]. Patients receiving venovenous or venoarterial ECMO have traditionally been considered too unstable for active physical therapy, frequently are heavily sedated, and occasionally are administered neuromuscular blocking agents. However, the ability to ambulate while receiving ECMO support has been facilitated by advances in extracorporeal technology and cannulation techniques [14-17]. Additionally, mobilization may be facilitated when ECMO allows for weaning from invasive mechanical ventilation [18]. Although patients receiving ECMO as bridge to transplantation (BTT) are obvious targets for early rehabilitation to maintain their transplant candidacy, those requiring ECMO as a bridge to recovery (BTR) from acute respiratory failure should theoretically benefit similarly from early mobilization, though there are few published reports of success in such populations [18-21]. We describe our center’s experience with a multidisciplinary approach to early physical therapy, including ambulation, in patients requiring ECMO as either BTR from acute respiratory failure or BTT in cases of end-stage lung disease.

## Material and methods

### Data collection

A retrospective analysis was performed on 100 consecutive patients receiving ECMO for refractory respiratory or cardiac failure in the Medical Intensive Care Unit (MICU) at New York-Presbyterian Hospital/Columbia University College of Physicians and Surgeons starting in April 2009 – the period after which our ECMO mobilization program began. These patients, a subset of all patients receiving ECMO at our institution, are managed by a multidisciplinary team who adhere to standardized management guidelines for anticoagulation, ventilation, sedation and physical therapy specific to the MICU ECMO population. They do not typically include immediate postoperative patients who have undergone lung or heart transplantation or other cardiothoracic surgeries, the majority of whom are managed separately in the cardiothoracic ICU. Baseline demographic data were collected on all patients, including age, Acute Physiology and Chronic Health Evaluation II score at time of admission to the MICU, diagnosis, the goal of ECMO therapy (BTT or BTR), tracheostomy status and ECMO configuration. Ventilatory status, oxygenation and ECMO settings were recorded before, during and after all physical therapy sessions.

BTT patients may, in general, be expected to have lower severity of illness scores than BTR patients because the more critically ill potential BTT patients would be less suitable for transplantation and, therefore, not receive ECMO as BTT. Likewise, BTT patients may receive ECMO support for a longer duration than BTR patients, depending on the availability of transplantable organs, which in turn could provide more opportunities for physical therapy. For these reasons, BTT and BTR patients were analyzed separately.

To record the highest level of mobilization achieved by patients receiving ECMO, the following ordinal scale was used: (1) no mobilization or passive range of motion of extremities, (2) turning in bed (including active-assisted range of motion of extremities), (3) sitting in bed with the head of bed elevated, (4) sitting on the edge of the bed with feet on floor, (5) out of bed sitting in a chair, (6) standing out of bed, (7) marching in place, and (8) ambulating (Table 1). This mobilization scale was adapted from an early version of a validated ICU Mobility Scale [22]. Outcomes include survival to transplant or discharge, discharge disposition among survivors (home, acute rehabilitation, subacute rehabilitation), and critical safety events that occurred during the course of physical therapy treatment sessions, including patient-related complications (hemodynamic instability, sustained desaturation to <85%, cardiac arrest, arrhythmia, bleeding at catheter site, new limb ischemia, non-ECMO catheter dislodgement) and circuit-related complications (oxygenator failure, pump failure, cannula dislodgement, tubing rupture, interruptions in blood flow).

**Table 1:** Mobilization scale characterizing level of activity in ECMO patients

**PT level**	**Level of activity**
1	No mobilization or passive range of motion of extremities
2	Turning in bed (including active-assisted range of motion of extremities)
3	Sitting in bed with the head of bed elevated
4	Sitting on the edge of the bed with feet on floor
5	Sitting in a chair
6	Standing
7	Marching in place
8	Ambulation

ECMO, extracorporeal membrane oxygenation; PT*,* physical therapy. Adapted from an early version of the validated ICU Mobility Scale [22].

### Statistical analysis

We summarized data using means with standard deviations, medians and ranges, or proportions. The chi-square test was used to determine statistical difference between categorical variables. All statistical analyses were performed with Microsoft Excel, version 14.3.2.

### Multidisciplinary approach to physical therapy and early mobilization

**Figure 1:**
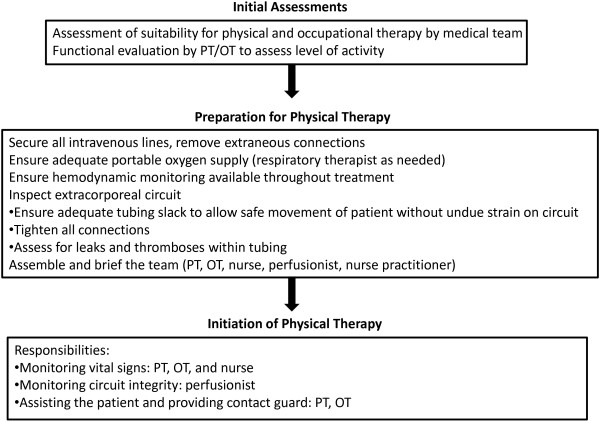
**Multidisciplinary, stepwise approach to physical therapy in the medical intensive care unit.** PT, physical therapist; OT, occupational therapist.

All ECMO recipients are evaluated daily from Monday through Saturday for their suitability for participation in physical and occupational therapy, which includes assessments by physical and occupational therapists, nurses, nurse practitioners, respiratory therapists and intensive care physicians. Reasons for deferring therapy, assessed on an individual basis and at the discretion of the treatment team, include clinically significant hemorrhage, unstable arrhythmia, severe thrombocytopenia, hemodynamic instability requiring high-dose vasopressors, severe hypoxemia despite oxygen supplementation, sedation precluding active participation by the patient and use of neuromuscular blockade. The mobilization team consists of a physical therapist, a perfusionist and a critical care registered nurse, with or without a critical care nurse practitioner, a respiratory therapist or an ECMO intensivist or surgeon, depending on the functional needs and clinical stability of the patient. The minimum number of participating clinicians is three. We use a simplified ECMO circuit that includes only the core components (centrifugal pump, membrane oxygenator, console and tubing). All other intravenous therapies, including hemodialysis, are administered through separate access points. Nonessential therapies are temporarily discontinued during physical therapy sessions (Figure 1). Particular attention is paid to the integrity of the ECMO cannula and tubing. A stabilization device, consisting of thermoplastic splinting material, may be used to secure the ECMO cannula and tubing during mobilization (An additional image file demonstrates the use of this device in more detail [see Additional file 1]). The ECMO circuit, consisting of a centrifugal pump, polymethylpentene oxygenator and console, is kept on a sprinter cart that can be mobilized alongside the ambulatory patient. During physical therapy sessions, support for gas exchange, in the form of ECMO sweep gas flow rates, ECMO blood flow rates and supplemental oxygen, may all be increased as needed, based on clinician judgment. Hemodynamic and respiratory statuses are monitored throughout, including the use of continuous pulse oximetry. Physical therapy sessions may be interrupted or terminated, based on the judgment of the participating clinicians, for changes that include hemodynamic instability, hypoxemia, dizziness, weakness, chest pain or dyspnea.

This study was conducted in accordance with the amended Declaration of Helsinki. The Columbia University Institutional Review Board approved the study (IRB Committee: Exp; Approval # IRB-AAAF3940). All necessary consents for patients involved in the study were obtained.

## Results

### Baseline demographics

Among 100 consecutive patients managed with ECMO for refractory respiratory or cardiac failure in the MICU starting in April 2009, 26 patients received ECMO with the intention of BTT, and 74 patients received ECMO as BTR. Thirty-five of the 100 patients participated in active physical therapy while receiving ECMO support (Table 2). Six BTT patients (32%) and 12 BTR patients (75%) had primarily hypoxemic respiratory failure. Seven BTT patients (37%) and four BTR patients (25%) had primarily hypercapnic respiratory failure. Five BTT patients (26%) had combined hypoxemic and hypercapnic respiratory failure. Pre-ECMO arterial blood gas analyses demonstrated marked impairment in gas exchange (Table 2). A high severity of illness was also evident in the mean Acute Physiology and Chronic Health Evaluation (APACHE) II score of 24.3 ± 7.8 in the total cohort of patients participating in active physical therapy, with a particularly high APACHE II score, 28.8 ± 7.8, in the BTR subset.

**Table 2:** Baseline demographics of ECMO patients undergoing active physical therapy in the MICU

	**Total (n = 35)**	**BTT (n = 19)**	**BTR (n = 16)**
Age (mean ± SD)	45.2 ± 18.7	43.4 ± 13.2	47.4 ± 23.9
Female (n, %)	20 (57)	11 (59)	9 (56)
Diagnosis (n, %)			
CF	10 (29)	10 (53)	0
ARDS	9 (26)	NA	9 (56)
ILD	6 (17)	6 (32)	0
COPD	6 (17)	2 (11)	4 (25)
PAH	4 (11)	1 (5)	3 (19)
APACHE II (mean ± SD)	24.3 ± 7.8	20.4 ± 5.8	28.8 ± 7.5
Pre-ECMO PaO_2_: FIO_2_ ratio in hypoxemic patients (mm Hg, mean ± SD)	58.3 ± 13.1	62.0 ± 12.2	55.0 ± 13.5
Pre-ECMO pH in hypercapnic patients (mean ± SD)	7.21 ± 0.13	7.24 ± 0.10	7.15 ± 0.17
Pre-ECMO PaCO_2_ in hypercapnic patients (mean ± SD)	96.3 ± 27.2	105.5 ± 27.9	81 ± 19.3
Venovenous ECMO via dual-lumen catheter (n, %)	23 (66)	14 (74)	9 (56)
Venoarterial ECMO via subclavian artery and internal jugular vein (n, %)	4 (11)	3 (16)	1 (6)
Femoral cannulation (n, %)	8 (23)	2 (11)	6 (38)

APACHE II, Acute Physiology and Chronic Health Evaluation II; ARDS, acute respiratory distress syndrome; BTR, bridge to recovery; BTT*,* bridge to transplant; CF, cystic fibrosis; COPD, chronic obstructive lung disease; ECMO, extracorporeal membrane oxygenation; FIO_2_, fraction of inspired oxygen; ILD, interstitial lung disease; MICU, medical intensive care unit; NA, not applicable; PaCO_2_*,* arterial partial pressure of carbon dioxide; PAH*,* pulmonary arterial hypertension; PaO_2_, arterial partial pressure of oxygen; SD, standard deviation.

### ECMO support

The mean ECMO blood flow rates and sweep gas flow rates both before and during physical therapy sessions are detailed in Table 3. The fraction of oxygen delivered through the ECMO circuit was maintained at 1.0 for all patients until they were ready to wean from ECMO.

**Table 3:** ECMO settings, ventilatory status and vasopressor requirements

	**Total (n = 35)**	**BTT (n = 19)**	**BTR (n = 16)**
ECMO blood flow rate pre-PT (LPM, mean ± SD)	2.99 ± 0.88	2.99 ± 0.81	3.00 ± 0.99
ECMO blood flow rate during PT (LPM, mean ± SD)	2.97 ± 0.94	3.02 ± 0.82	2.92 ± 1.09
ECMO sweep gas flow rate pre-PT (LPM, mean ± SD)	2.97 ± 1.79	3.45 ± 1.71	2.39 ± 1.77
ECMO sweep gas flow rate during PT (LPM, mean ± SD)	2.96 ± 1.80	3.46 ± 1.71	2.35 ± 1.78
Tracheostomy on ECMO (n, %)	11 (31)	9 (47)	2 (13)
Successful liberation from invasive mechanical ventilation while receiving ECMO (n, %)	23 (66)	13 (68)	10 (63)
Room air (n, %)	3 (13)	2 (15)	1 (10)
Nasal cannula (n, %)	17 (74)	8 (62)	9 (90)
High flow nasal cannula (n, %)	3 (13)	3 (23)	0
Vasopressor use during PT (n, %)	9 (26)	5 (26)	4 (25)
Dose of norepinephrine (mcg/min, median, IQR)	2 (0.5 to 5)	3.5 (1.3 to 5)	1.3 (0.5 to 2)
Dose of vasopressin (units/min)	0.04	0.04	0.04

BTR, bridge to recovery; BTT*,* bridge to transplant; ECMO, extracorporeal membrane oxygenation; LPM, liters per minute; IQR, interquartile range; PT, physical therapy.

### Invasive mechanical ventilation status and supplemental oxygen requirements

Two-thirds of participants in physical therapy were liberated from invasive mechanical ventilation while receiving ECMO, with similar frequency in both BTT and BTR groups (Table 3). Three patients were weaned to no oxygen support, seventeen patients were supported with conventional nasal cannula, and three patients were supported with high flow nasal cannula. From their first through last physical therapy sessions while receiving ECMO, three patients had an overall decrease in the amount of conventional ventilatory support, three patients required more support, and the remaining 29 patients had no change in the amount of support needed. For individual physical therapy sessions, there were no differences between the amount of support needed before and during therapy. Approximately one-third of patients received a tracheostomy while receiving ECMO, with a higher percentage of BTT patients undergoing tracheostomy than BTR patients.

### Physical therapy

The median maximum physical therapy score achieved was 8 (interquartile range (IQR) 2 to 8) in the entire cohort, with a median of 8 in the BTT group and a median of 2 in the BTR group (Table 4). The maximal level of activity achieved was bed-level active-assisted range of motion in eleven patients (32%), sitting in bed in two patients (6%), sitting at the edge of the bed in one patient (3%), standing in three patients (9%), and ambulating in eighteen patients (51%). The median number of physical therapy sessions per patient was five, with a median of 2.8 sessions per patient per week. Among the eighteen patients who achieved ambulation, the median distance walked was 175 feet (IQR 37.5 to 285), with one BTT patient able to use an in-bed restorator bicycle for 4 minutes, a second patient able to ambulate 1,600 feet and use a bedside stationary bicycle for 25 minutes, and another patient able to ambulate unassisted up to 2,800 feet daily. One patient with a femoral venous ECMO catheter was able to stand with minimal assistance, and a second patient with a femoral venous ECMO catheter was able to ambulate 4 feet. Thirteen patients improved their physical therapy score over the course of their time on ECMO, nineteen patients maintained the same level of activity, and three patients had a decline in their physical therapy score.

**Table 4:** Physical therapy, survival and discharge data

	**Total (n = 35)**	**BTT (n = 19)**	**BTR (n = 16)**
Maximum PT score (median, IQR)	8 (2 to 8)	8 (6 to 8)	2 (2 to 8)
No. of PT sessions per patient (median, IQR)	5 (1 to 13)	13 (8 to 15)	1.5 (1 to 3.25)
No. of PT sessions/patient/week (median, IQR)	2.8 (0.5 to 7.8)	4.5 (1.4 to 7.8)	1.3 (0.5 to 6.4)
Time from initiation of ECMO to first PT session (days, median, IQR)	2 (1 to 4.5)	2 (1 to 2)	4 (1.75 to 5.75)
No. of ambulatory patients (n, %)	18 (51)	12 (63)	6 (38)
Maximum distance ambulated (ft, median, IQR)	175 (37.5 to 285)	170 (55 to 525)	195 (60 to 398)
Survival to transplantation (n, %)	NA	10 (53)	NA
Survival to discharge (n, %)	23 (66)	9 (90)^a^	14 (88)
Disposition of survivors (n, %)			
Home	13 (57)	4 (44)	9 (64)
Acute rehabilitation	8 (35)	4 (44)	4 (29)
Subacute rehabilitation	2 (9)	1 (11)	1 (7)

^a^Among those patients who survived to transplant. BTR, bridge to recovery; BTT, bridge to transplant; IQR, interquartile range; PT, physical therapy.

Compared with ECMO settings before physical therapy, there was no overall difference in mean ECMO blood flow rates or sweep gas flow rates before, during, or after all physical therapy sessions (Table 3). One patient who ambulated 1,600 feet and used the cycle ergometer had his ECMO blood flow rate consistently increased by 0.5 LPM for all physical therapy sessions without adjustment of sweep gas flow rates. Eight patients (24%) required an increase in the amount of supplemental oxygen during physical therapy to maintain adequate oxygenation, seven of whom were ambulatory.

Twenty-six percent of patients were receiving vasopressors at the time of physical therapy (Table 3). No adjustments were made to vasopressor doses during physical therapy.

### Outcomes

The mean duration of ECMO in the BTT patients who participated in active physical therapy was 18.7 ± 13.2 days. Ten of the BTT patients (53%) survived to lung transplantation, with a mean duration of ECMO of 13.8 ± 7.8 days prior to transplant. Nine of those patients remain alive post-transplant at the time of this report, with one patient having died 29 days post-transplant from multi-organ failure. All but one of the transplant survivors were discharged directly to home or admitted to acute rehabilitation facilities prior to discharge to home (Table 4). For those patients who survived to discharge, the mean hospital length of stay post-transplant was 33.6 ± 10.9 days.

The mean duration of ECMO in the BTR group was 9.1 ± 2.6 days, with 88% surviving to decannulation and subsequent discharge. All but one of the BTR survivors were discharged directly to home or admitted to acute rehabilitation facilities prior to discharge to home. The mean hospital length of stay post-ECMO decannulation was 17.9 ± 17.2 days.

### Trends in participation in physical therapy

**Figure 2:**
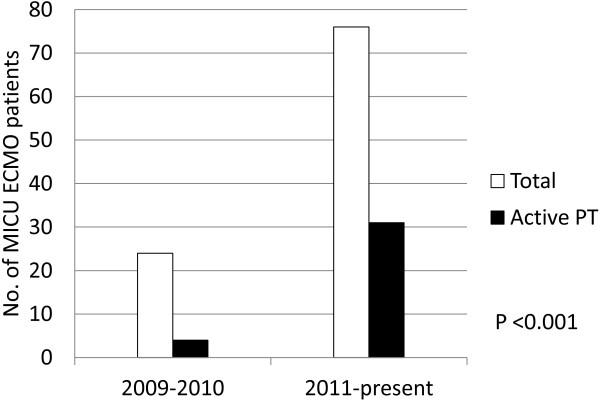
**Trends in active physical therapy participation by ECMO patients in the MICU over time.** ECMO extracorporeal membrane oxygenation; MICU Medical Intensive Care Unit; PT physical therapy.

Compared with the rate of participation in active physical therapy among MICU ECMO patients from 2009 to 2010, there has been a significantly higher percentage of patients participating in active physical therapy since 2011 (17% versus 41%, *P* <0.001) (Figure 2).

### Complications

There were no patient-related or circuit-related complications as a result of physical therapy treatment sessions in any of the patients.

## Discussion

There is increasing evidence demonstrating improved outcomes from early rehabilitation, including ambulation, in critically ill patients [1,7-10]. With the increasing use of ECMO for patients with either acute, potentially reversible, respiratory failure or end-stage lung disease awaiting transplantation, there is a growing need to evaluate the feasibility, safety, and functional outcomes of performing physical rehabilitation and early mobilization within this patient population.

Previously published case series describing attempts at active physical therapy and mobilization in patients receiving ECMO have reported small cohorts with varying degrees of success, and these were mostly pre-transplant patients [18-21,23-27]. In order to maximize the success of physical therapy and minimize the risk of complications, we have developed a multidisciplinary team approach to physical therapy for patients receiving ECMO.

Older generations of extracorporeal technology were cumbersome and difficult to mobilize. Additionally, traditional ECMO configurations involved femoral cannulation, making mobilization, especially ambulation, a high-risk intervention because ECMO catheters are larger and less flexible than other catheters typically used in medical intensive care [28] and dislodgment has more severe consequences. Despite these concerns, we were successful in achieving standing or ambulation in two patients with femoral venous ECMO cannulae, demonstrating feasibility. The risks associated with mobilization while receiving ECMO have been lessened by the concurrent development of more compact circuitry and dual-lumen catheters that can provide venovenous extracorporeal support via an upper-body approach [14,15,29]; however, these dual-lumen cannulae require particular expertise for proper placement and are associated with complications unique to this approach [30]. Additionally, the combination of internal jugular venous and subclavian arterial cannulation permits venoarterial ECMO support via an upper-body configuration [17]. Our experience suggests that while femoral cannulation is not an absolute contraindication to ambulation, it is certainly not optimal, and an upper-body configuration is our recommended approach to facilitate ambulation and avoid cannula complications during mobilization.

With increases in extracorporeal gas exchange efficiency, select patients are eligible for endotracheal extubation while receiving ECMO. Extubation eliminates the accumulation of complications associated with invasive mechanical ventilation (ventilator-associated pneumonia, ventilator-associated lung injury, dynamic hyperinflation, impaired delivery of aerosolized medications), reduces the need for sedatives commonly employed to maximize patient-ventilator synchrony, improves patient comfort, and further facilitates ambulation by having one less apparatus to mobilize, all of which may contribute to improved outcomes [31-35]. We were successful in liberating 66% of the ECMO patients receiving active physical therapy from invasive mechanical ventilation, with similar rates in both BTT and BTR groups. Liberation from invasive mechanical ventilation occurred in parallel with the physical therapy program.

Our ability to safely and repeatedly mobilize patients being bridged to either transplant or recovery was demonstrated despite a high severity of illness, as evidenced by very severe pre-ECMO gas exchange abnormalities and high APACHE II scores. These scores may underestimate the true severity of illness in patients transferred from outside institutions due to missing data representing the most severe physiologic derangements prior to the initiation of ECMO. Patients were able to engage in active physical therapy despite the need for low-dose vasopressors, and no significant changes in ECMO settings were needed during activity with the exception of one patient who engaged in cycling. Physical therapy sessions and ambulation were more frequent in the BTT group, which may be explained, in part, by the longer duration of ECMO (18.7 versus 9.1 days), lower average APACHE II score (20.4 versus 28.8), lower percentage of patients with hypoxemic respiratory failure (58% versus 75%) and, to a much lesser extent, less severe hypoxemia (PaO_2_:FIO_2_ of 62 versus 55) in the BTT group than the BTR group. As expected, BTT patients had lower severity of illness scores than BTR patients, in part because those who are too critically ill may be unsuitable for transplantation and, therefore, not considered candidates for ECMO as BTT.

Despite the advances in extracorporeal technology, not all ECMO patients may be appropriate candidates for early rehabilitation. Overall, approximately one third of our total ECMO cohort was able to participate actively in physical therapy, although our rate of participation has increased by more than 240% from 2009 to the present, with more than 40% of recent ECMO patients participating in active physical therapy. The increased rate is due, in part, to increased experience and comfort with mobilizing critically ill patients in general, which was aided by the initiation of a global early mobilization program in our MICU.

Over the course of the study period, we relaxed our criteria for participation in rehabilitation, in parallel with increasing comfort by our staff in treating patients with marked physiologic derangements. Patients on low-dose vasopressors or requiring high ventilatory support who, early in our experience, were deemed too unstable for mobilization are now more routinely engaged in physical therapy. However, there are clinical parameters that may limit physical therapy. Patients who are hemodynamically unstable requiring high-dose vasopressor support are not engaged in physical therapy. Additionally, those who are deeply sedated, receiving neuromuscular blockade, or severely hypoxemic despite extracorporeal support remain a population that is unsuitable for our mobilization protocol, and accounted for the vast majority of patients who did not participate in active physical therapy in our cohort. However, compared with the earlier study period, our approach to critically ill patients in general, and ECMO patients specifically, has evolved to incorporate less sedation and neuromuscular blockade, allowing more patients to remain awake during their MICU stay, accounting for the significant difference in patients participating in active physical therapy. Because the more critically ill patients, with a higher expected mortality, were the ones unable to participate in mobilization, comparison of outcomes between those who did and did not receive active physical therapy would not reflect just the impact of our protocol on those outcomes. Larger, prospective studies are needed to better define the barriers to physical therapy and mobilization in ECMO patients and to understand the effect of these interventions on functional outcomes. Future studies may also help establish protocols regarding adjustments to ECMO settings as necessary to compensate for increases in activity that can be standardized and applied across different ECMO patient populations. Cost-benefit analyses are also warranted given the cost associated with increased staffing needs for early mobilization protocols. Although occupational therapy statistics are not represented in this study, it would be beneficial for future studies to examine the impact of occupational therapy on the ECMO population. Occupational therapists are now participating in the early mobilization of these patients with a focus on activities of daily living, upper extremity strengthening and coordination, cognition and communication.

## Conclusions

Active physical therapy, including early mobilization, may be performed in patients receiving ECMO. Advances in technology and a multidisciplinary team approach facilitate the safe and reliable implementation of such an intervention, even among patients with a very high severity of illness. Additional research is needed to characterize the long-term functional, neurocognitive and psychiatric impact of physical and occupational therapy on patients receiving ECMO.

## Key messages

• Active physical therapy is safe and feasible for patients receiving ECMO.

• A multidisciplinary team approach facilitates the implementation of a rehabilitation program for ECMO recipients.

• Both patients awaiting transplantation and those being bridged to recovery may benefit from active physical therapy, although the optimal patient population remains to be defined.

• Future studies are needed to better define barriers to physical therapy in ECMO recipients and whether such interventions have a favorable impact on major clinical outcomes and are cost effective.

## Abbreviations

APACHE: Acute Physiology and Chronic Health Evaluation; BTR: bridge to recovery; BTT: bridge to transplant; ECMO: extracorporeal membrane oxygenation; FIO_2_: fraction of inspired oxygen; LPM: liters per minute; MICU: medical intensive care unit; PaCO_2_: partial pressure of carbon dioxide in arterial blood; PaO_2_: partial pressure of oxygen in arterial blood; PT: physical therapy.

## Competing interests

Dr. Brodie reports receiving research support from Maquet Cardiovascular including travel expenses for research meetings, research support for the present study as well as anticipated support for upcoming studies and compensation paid to Columbia University for research consulting. He receives no direct compensation from Maquet. He is a member of the Medical Advisory Board for ALung Technologies. Compensation is paid to Columbia University; he receives no direct compensation from ALung Technologies.

Dr. Bacchetta reports receiving research support from Maquet Cardiovascular including travel expenses for research meetings, research support for the present study as well as anticipated support for upcoming studies and compensation paid to Columbia University for research consulting. He receives no direct compensation from Maquet.

The remaining authors declare that they have no competing interests.

## Authors’ contributions

DA, JJ, CA, MB and DB contributed to the conception and design, acquisition of data, analysis and interpretation of data; EF, LM, DZ, KG and TM contributed to the acquisition of data; PR and PB contributed to the analysis and interpretation of data. All authors (DA, JJ, EF, LM, CA, DZ, KG, TM, PR, PB, MB, DB) were involved in drafting the manuscript or revising it critically for important intellectual content. All authors read and approved the final manuscript.

## Authors’ information

Co-senor authors are Matthew Bacchetta and Daniel Brodie.

## Supplementary Material

Additional file 1 **Cannula stabilization device.** Description: Photo of the cannula stabilization device (referred to as the ‘snorkel’) used to secure cannula and tubing during mobilization. Left Panel: Stand-alone device. Right Panel: Demonstration of device in use. Invented by David Zemmel. Click here for file
