# Supplementary figures and images for: Early mobilization of patients receiving extracorporeal membrane oxygenation: a retrospective cohort study
Source: Crit Care. 2014 Feb 27;18(1):R38. doi: 10.1186/cc13746 (PMC4056162; doi:10.1186/cc13746)

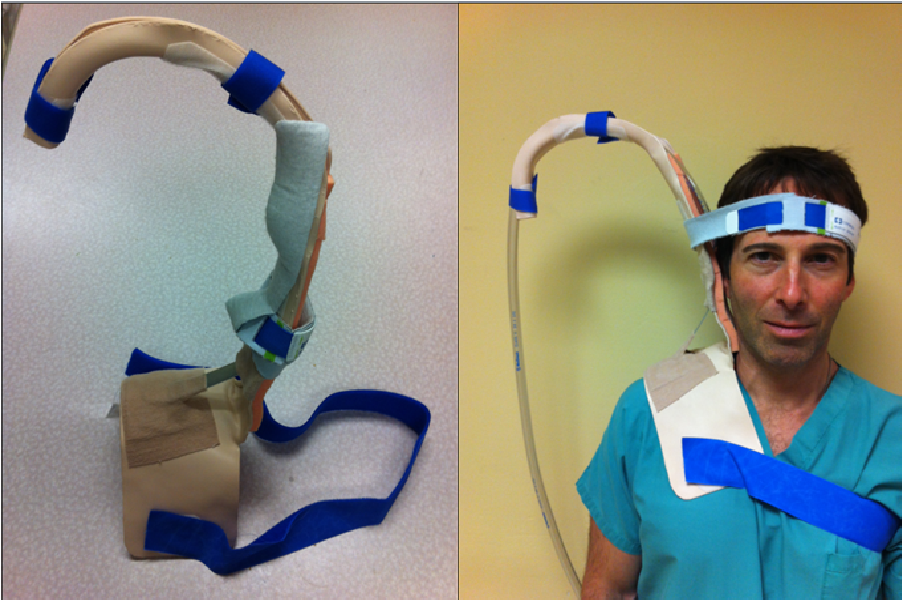

Supplement: Additional file 1 — Cannula stabilization device. Description: Photo of the cannula stabilization device (referred to as the ‘snorkel’) used to secure cannula and tubing during mobilization. Left Panel: Stand-alone device. Right Panel: Demonstration of device in use. Invented by David Zemmel. [file cc13746-S1.tiff]
